# Monitoring Intrarenal Pressure vs. Irrigation Fluid Absorption for Infection Prevention in Ureteroscopy

**DOI:** 10.1155/aiu/3521554

**Published:** 2026-07-24

**Authors:** Guilin Wang, Yutong Lu, Zhichun Yang, Jiangtao Bai, Jun Mi, Qiqi He, Zhiping Wang

**Affiliations:** ^1^ Department of Urology, The Second Hospital of Lanzhou University, Lanzhou, China, lzu.edu.cn; ^2^ Gansu Province Clinical Research Center for Urinary System Disease, Lanzhou, China

**Keywords:** infection, intrarenal pressure, irrigation fluid absorption, monitoring, ureteroscopy

## Abstract

As ureteroscopy (URS) is increasingly utilized, preventing and managing severe postoperative infections—such as urosepsis—has become a clinical priority. Elevated intrarenal pressure (IRP) and irrigation fluid absorption (IFA) represent two core pathophysiological steps in the translocation of bacteria and endotoxins into the bloodstream. This review compares the clinical utility and technological maturity of IRP and IFA monitoring during URS. The analysis indicates that IRP is the instantaneous physical driving force for pyelovenous backflow, whereas IFA is the integrated outcome of multidimensional variables, including the pressure gradient, operative time and tissue permeability. Consequently, monitoring a single dimension may be insufficient to comprehensively assess infection risk. IRP monitoring (e.g., using a ureteral access sheath with a pressure‐sensing channel or a flexible URS with an integrated pressure sensor) offers the advantage of real‐time feedback, potentially playing a key role in maintaining low IRP and proactively preventing intrarenal backflow. However, it cannot account for the cumulative effect of time or changes in tissue permeability. IFA monitoring, despite its inherent lag, may provide a more comprehensive quantitative measure of the pathogenic load absorbed by the patient, suggesting potential predictive and warning value for the occurrence of infection; however, clinically validated thresholds and prospective evidence are currently lacking. In the future, with advancements in monitoring technology, the development of automated closed‐loop systems integrating high‐precision IRP monitoring, real‐time IFA monitoring, intelligent irrigation and negative pressure suction holds promise for maximizing the balance between surgical efficacy and patient safety, provided that future studies can establish evidence‐based intervention triggers.

## 1. Introduction

Nephrolithiasis prevalence is rising, with five‐year recurrence rates of 20%–50%, representing a growing public health burden [1]. Ureteroscopy (URS) use has increased dramatically. Overall postoperative complication rates range from 9% to 25%, with most being minor [2]; however, infectious complications—fever, systemic inflammatory response syndrome (SIRS) and urosepsis—remain a significant challenge.

Beyond traditional risk factors (positive preoperative urine culture, urine leukocytosis, large stone burden, prolonged operative time, female sex and diabetes mellitus), elevated intrarenal pressure (IRP) and irrigation fluid absorption (IFA) are increasingly recognized as independent risk factors for post‐URS infection. During URS, continuous irrigation maintains visibility, but instruments elevate IRP. When IRP exceeds a threshold, pyelovenous backflow transports bacteria‐laden irrigation fluid into the bloodstream, increasing infection risk. IRP drives IFA: in a mini‐percutaneous nephrolithotomy (PCNL) model, for every 1 mmHg increase in mean IRP, the absorption rate increases by 54.2 mL/h [3] and absorbed volume itself is an independent risk factor for postoperative infectious fever in mini‐PCNL [4]. Peak IRP during flexible URS can reach 289.3–436.9 cmH_2_O [5]. In a porcine model, IRP maintained at 20–70 mmHg for 30–60 min caused intrarenal backflow spread throughout the renal cortex; backflow was visible even at 20 mmHg [6]. In a porcine URS model, procedures at 75 mmHg and 150 mmHg produced significantly higher IFA volumes and rates compared with retrograde URS at 37 mmHg [7]. Irrigating with saline containing *Escherichia coli* at 75 mmHg in a porcine URS model yielded significantly higher bacteraemia incidence and inflammatory biomarkers than the 37 mmHg group or controls [8]. Furthermore, in a porcine model, IRP of 30–120 mmHg induced bacterial sepsis and *E. coli* concentration in pyelovenous backflow increased exponentially above 60 mmHg [9].

Vacuum suction ureteral access sheaths and intelligent pressure control systems have been introduced, and studies confirm they significantly reduce postoperative fever and urosepsis by actively lowering or dynamically regulating IRP [10–12]. However, relying solely on IRP has limitations. IRP is the physical driving force; IFA is the direct vehicle for bacteria and endotoxins entering the bloodstream. Thus, a comprehensive assessment based on IFA kinetics may better reflect the bacterial load and endotoxin challenge. IFA is influenced not only by IRP but also by irrigation duration, mucosal injury and individual anatomical differences—factors themselves independent of infection risks. During stone fragmentation, bacteria and endotoxins released from calculi become suspended in irrigant; IFA implies direct systemic absorption of these pathogenic substances.

Both IRP and IFA monitoring possess distinct value. IRP monitoring is simpler, more immediate, and more accurate, lending itself to infection prevention. IFA may better reflect absorption kinetics and holds potential for predicting and assessing post‐URS infection, although its clinical utility requires further validation. This Review compares IRP and IFA monitoring during URS, delving into their mechanistic connections, fluid‐dynamic differences, clinical value, and future technological directions.

## 2. Review Methodology

This is a narrative review focusing on synthesizing evidence from clinical studies, animal models and in vitro kidney models regarding IRP and IFA during URS, with the aim of comparing the clinical value of IRP monitoring and IFA monitoring for infection prevention. We performed a comprehensive literature search of PubMed, Web of Science and Embase databases from inception through December 2025. The search strategy included the following keyword combinations: (“ureteroscopy” OR “flexible ureteroscopy” OR “retrograde intrarenal surgery”) AND (“intrarenal pressure” OR “renal pelvic pressure” OR “intrapelvic pressure” OR “intrarenal pelvic pressure” OR “renal pelvis pressure”); (“ureteroscopy” OR “flexible ureteroscopy” OR “retrograde intrarenal surgery”) AND (“irrigation fluid absorption” OR “fluid absorption” OR “fluid extravasation” OR “pyelovenous backflow”). After removing duplicates, a total of 258 records were retrieved, including 177 original articles comprising clinical studies, animal experiments and in vitro kidney model studies. In addition, considering that URS and other related endoscopic procedures—particularly PCNL—may share partly overlapping pathophysiological mechanisms, monitoring technologies or clinical outcomes regarding IRP and IFA, literature on PCNL, transurethral resection of the prostate (TURP), and other studies on IRP and backflow was also retrieved. Studies involving infection were prioritized. Owing to the large volume of literature, for studies addressing the same or similar topics, we cited only a selection of high‐quality, representative, or the most recently published articles, chosen based on the authors’ professional judgment. For evidence derived from non‐URS procedures (e.g., mini‐PCNL and TURP), the source is explicitly indicated throughout the manuscript, and appropriate caution is exercised when extrapolating such findings to URS.

## 3. From Pressure Drive to Absorbed Load: A Mechanistic Analysis

Understanding post‐URS infection pathogenesis requires distinguishing the fluid dynamic driving force (pressure) and the pathogenic load effect (absorption).

### 3.1. The Fluid Dynamic Driving Role of Intrarenal Pressure

During URS, IRP is influenced by UAS size, negative pressure suction, forced irrigation, irrigation pressure, and stone location. Physiological IRP is 0–15 mmHg [13]. Larger UAS sizes (13/15 Fr) yield significantly lower baseline, median and maximum IRP versus smaller (11/13 Fr): 5 mmHg vs. 18.5 mmHg, 21 mmHg vs. 57 mmHg and 111 mmHg vs. 177.5 mmHg in a clinical URS study [14]. Negative‐pressure UAS (NP‐UAS) lower IRP more effectively than traditional UAS, with pressure decreasing as suction negative pressure increases [15, 16]. With a UAS and irrigation pressure set at 40 cmH_2_O (1 cmH_2_O = 0.735561 mmHg), median IRP without forced irrigation was 34 (19–81.6) cmH_2_O; during laser lithotripsy, median IRP with and without forced irrigation was 61.2 (27.2–149.5) cmH_2_O; peak IRP during forced irrigation with lasering reached 54.4–236.6 cmH_2_O [17]. With the endoscope in the renal pelvis and irrigation pressure 80 cmH_2_O without forced irrigation, mean IRP was 63 cmH_2_O; on‐demand forced irrigation raised mean IRP to 115.3 cmH_2_O, with peaks 289.3–436.9 cmH_2_O [5]. In a randomized clinical trial of URS, manual hand pump irrigation produced higher mean (62.29 vs. 38.16 mmHg) and peak (192.71 vs. 68.04 mmHg) IRP compared to a 100 mmHg pressure bag [18].

Primary direct consequences of elevated IRP include intrarenal fluid backflow (absorption), renal parenchymal injury and anatomical disruption [13, 19, 20]. If bacteria are present, backflow transports bacteria and toxins into the bloodstream, potentially causing bacteraemia or sepsis [21]. When IRP exceeds∼30 mmHg, the pressure gradient forces fluid across barriers via pyelotubular, pyelolymphatic, pyelovenous backflow and structural extravasation [21]. The IRP threshold for backflow is lower than for parenchymal injury but exhibits individual variability. Contrast‐enhanced ultrasound during mini‐PCNL showed that exceeding ∼34 mmHg induced pyelotubular backflow and reduced renal cortical perfusion [22]. Gadolinium‐enhanced MRI in a porcine model showed the mean pressure for first visual gadolinium reflux into the renal cortex was 21 mmHg, and the level of backflow was a function of both IRP and time [6]. Thresholds for different backflow pathways vary: pyelotubular backflow occurs first, then pyelolymphatic, then pyelovenous [23, 24]. In an isolated perfused porcine kidney, collecting duct involvement and contrast entry into supporting tissue occurred at 5 mmHg, while venous involvement appeared at 30 mmHg [24]. During most URS procedures, pressure peaks far exceeding these thresholds are frequently reached. IRP is the initiating factor; without a sufficient pressure gradient, backflow does not occur.

### 3.2. The Volume Carrier Effect of IFA

IFA is the quantitative transfer of a mixture containing bacteria, endotoxins, and inflammatory mediators into the systemic circulation, serving as the direct pathogenic vector for SIRS and urosepsis. Its anatomical pathways are identical to those of intrarenal backflow. In an early URS clinical study, using the volumetric balance method, mean estimated fluid absorption during URS was 54 mL (range 4–137 mL) [25]. In another clinical study, blood ethanol measurements showed IFA during URS ranged from 20 to 573 mL, with some absorption in all patients [26]. IFA volume is influenced by IRP, irrigation duration, irrigant type, anatomy and kidney disease, with pressure and time being the most significant modifiable risk factors [6, 27]. Typically, small volumes pose low risk of electrolyte disturbance and fluid overload, but infectious complications and renal tissue injury remain significant concerns.

IFA critically facilitates translocation of bacteria and endotoxins from the urinary tract to the bloodstream. Calculi often harbour bacteria as biofilms [28]; laser lithotripsy releases viable bacteria and high concentrations of endotoxins from the stone matrix [29, 30]. When IRP drives backflow, these are carried into venous circulation. IRP provides motive force; IFA determines the quantity absorbed. Theoretically, absorbed pathogen quantity equals the product of absorbed fluid volume and the average concentration of released substances. Sole pressure monitoring cannot quantify the actual pathogenic load; therefore, monitoring IFA may offer a more comprehensive assessment from the volume carrier perspective, though this concept remains primarily hypothesis‐generating based on mechanistic principles rather than direct clinical validation.

### 3.3. Pressure‐Driven Absorption Mechanisms: Why Pressure Control Alone Is Insufficient

IFA is not solely driven by IRP but co‐regulated by time and the biological barrier state. We propose a conceptual IFA kinetics model:

(1) QIFA=∫0TK A Prenalt−Pvenoustdt,

where *Q*_IFA_ is the total volume of irrigation fluid absorbed, *T* is the total irrigation duration, *K* is the tissue permeability coefficient, *A* is the absorption surface area and *P*_renal_(*t*) and *P*_venous_(*t*) are the renal pelvic and venous pressures at time *t*, respectively. According to this model, the cumulative absorbed fluid volume (*Q*_IFA_) is not simply determined by the IRP at any given moment (*P*_renal_(*t*)), but is the mathematical integral result of the interplay between the effective pressure gradient (*P*_renal_(*t*) − *P*_venous_(*t*)), tissue permeability (*K*), absorption area (*A*) and irrigation time (*t*).

This kinetic model adheres strictly to fluid dynamics theory and aligns with evidence from relevant clinical and animal studies. Each variable accurately maps to known risk factors during the surgical procedure. Firstly, IRP and time are the most widely studied variables. IRP primarily influences the rate of fluid absorption, as shown in a mini‐PCNL study [3]. The inclusion of the time variable illuminates the clinical reality of a cumulative effect: even if IRP is only slightly above the threshold, a sufficiently long procedure time dealing with an infected stone can allow this sustained low‐level absorption to accumulate, via time integration, into a significant and potentially harmful bacterial and volume load. Secondly, traditional research has paid less attention to the influence of tissue permeability (*K*) and effective absorption area (*A*). In this model, *K* directly reflects the anatomical integrity and patency of the renal pelvic mucosa and renal parenchymal barrier during URS. It is theoretically plausible that both *K* and *A* are dynamic during the procedure. Indirect evidence for changes in effective absorption area (*A*) comes from observations that the number of renal units involved in intrarenal backflow following an IRP increase varies between individuals [23, 24]. During URS, high IRP, thermal injury from lasering, and mechanical friction from the movement of guidewires and the access sheath can all lead to mucosal tears or microscopic disruptions of venous sinuses. These events would increase the permeability coefficient (*K*) and potentially the effective absorption area (*A*). In such a state, even relatively low IRP might be sufficient to drive irrigation fluid, laden with high concentrations of bacteria and endotoxins, into the circulation.

Sole IRP monitoring assesses only one variable. It cannot capture the cumulative amplification of time (*T*) or increased permeability (*K*) and absorption area (*A*) from injury. IRP provides an instantaneous snapshot; IFA is a cumulative outcome integrating IRP, time, tissue injury and individual differences. Theoretically, IFA may have greater explanatory power and comprehensiveness for infection pathophysiology than a single pressure metric, but this hypothesis remains to be tested in clinical studies.

## 4. Current Status and Limitations of Monitoring Technologies

In clinical practice, obtaining real‐time data on IRP and IFA has been a persistent technical challenge in endourology.

### 4.1. Intrarenal Pressure Monitoring Technologies

The development of IRP monitoring techniques has primarily focused on accurately measuring true hydrostatic pressure within the renal pelvis without interfering with the surgical procedure.

1. Percutaneous Nephrostomy Tube Manometry. Connecting a preplaced percutaneous nephrostomy (PCN) tube to an external pressure transducer allows real‐time IRP reading [31]. As most URS patients lack a PCN, this is largely confined to animal experiments or combined endoscopic procedures.
2. Retrograde Ureteral Catheter/Pressure Wire Manometry. A small‐diameter ureteral catheter or pressure guidewire is placed alongside the URS, its tip in the renal pelvis and proximal end connected to a transducer [5, 17, 32–34]. However, the additional catheter/wire occupies space within the ureter or UAS lumen, impeding endoscope manipulation and increasing outflow resistance, potentially causing artifactual IRP elevation.
3. Ureteral Access Sheath with a Pressure‐Sensing Channel. Specially designed sheaths incorporate an independent pressure‐sensing lumen opening at the sheath tip, with the external port connected to a transducer [10, 11, 35]. Advantage: does not occupy the effective inner diameter or alter routine practice. Limitation: the measurement point is in the proximal ureter or UPJ, not deep within a calyx. Based on Bernoulli’s principle, local velocity differences may cause discrepancy between sheath tip pressure and the calyceal pressure driving backflow; measured pressure is an approximation.
4. Flexible URS with Integrated Pressure Sensor. A miniaturized pressure sensor integrated into the endoscope tip enables precise measurement of true surrounding pressure [36, 37], resolving spatial occupation and measurement blind spots. Main drawback: high economic cost, preventing widespread adoption.

Data accuracy is paramount. Ureteral catheters and pressure‐sensing UASs rely on a fluid column to transmit pressure, requiring the renal pelvis and sensor to be at the same level; otherwise, a hydrostatic height difference creates error. Air bubbles and fluid inertia can affect accuracy and cause transmission delays [38]. Tip‐mounted sensors (pressure wires, integrated URS sensors) are the most accurate. Even accurate single‐point measurements may not reflect pressure compartmentalization: pressure elsewhere may be significantly higher or lower [39], and a device reading below the set reflux threshold does not guarantee safety throughout.

### 4.2. IFA Monitoring Technologies

Compared to measuring instantaneous pressure, quantifying IFA as a cumulative volume over time is more challenging. Unlike IRP monitoring, which has seen increasing clinical application, most IFA monitoring methods remain primarily research tools for various reasons, with the exception of one commercially available system for monitoring bleeding and fluid absorption in endoscopic surgery.

1. Volumetric/Gravimetric Balance Method. IFA = Total irrigant input–Total effluent output. Inexpensive but compromised by intraoperative bleeding, evaporation, drape soaking, spillage and urine output, leading to significant error [40, 41]; largely abandoned.
2. End‐tidal Ethanol Monitoring. Adding 1% sterile ethanol to the irrigation fluid as a tracer; ethanol crosses membranes and is exhaled. Continuously measuring exhaled ethanol concentration via a detector connected to the anaesthesia breathing circuit allows estimation of absorbed volume using pharmacokinetic formulas [27, 42–44]. Advantages: noninvasive, simple, high sensitivity. Limitations: reflects primarily vascular absorption and may lag for retroperitoneal uptake; inter‐individual ethanol metabolism variability, potential allergic reactions, and under general anaesthesia require special breathing circuit connections. Its noninvasive nature and good correlation with absorbed volumes make it a reference standard in many comparative studies.
3. Isotope‐ or Chemical‐Tracer Methods. Adding a radioactive isotope (e.g., Iodine‐131) or specific dye/ion to the irrigant and periodically sampling blood to measure tracer concentration [45, 46] (from TURP studies). High‐accuracy but radioactive materials, radiation protection, expensive equipment and cumbersome procedures prevent routine clinical application; they are used experimentally and for validation.
4. Haemodynamic and Electrolyte Indirect Monitoring. Detecting fluid absorption via intraoperative changes in central venous pressure, blood pressure or serum sodium on blood gas analysis [47, 48] (from TURP and PCNL studies). Requires blood sampling with delayed results; serum sodium changes lag and lack specificity. Significant volume overload or hyperchloraemic metabolic acidosis manifests only after substantial absorption.
5. Endoscopic Surgical Monitoring System (ESMS). A device for real‐time, noninvasive monitoring of IFA and blood loss during irrigation‐based endoscopic procedures [49]. ESMS uses load cells to continuously weigh the irrigant input and effluent output difference and a photoelectric sensor to measure haemoglobin concentration in effluent; a computer algorithm corrects for urine output to calculate blood loss and fluid absorption volume. Reported relative error for fluid absorption detection: 0.07%–1.00% [49]. ESMS is completely noninvasive, unaffected by individual metabolism, reflects total absorption (intravascular and extravascular), and provides real‐time, automated data. The limitation is the need to have the equipment.

## 5. Prevention vs. Prediction: A Comparison of Clinical Monitoring Value

**FIGURE 1:**
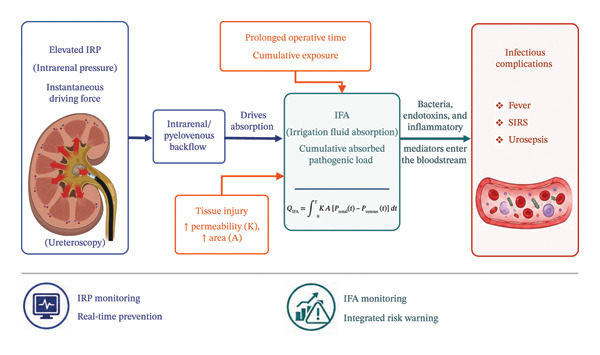
The relationship among intrarenal pressure, operative time, tissue injury, irrigation fluid absorption and infectious complications during ureteroscopy.

Based on the absorption kinetics model, IRP and IFA represent driving force and cumulative effect, respectively, leading to distinct monitoring roles: IRP for active prevention and IFA for sentinel warning (Table 1). A conceptual figure illustrating the relationship between IRP, operative time, tissue injury, IFA and infectious complications is proposed to aid clinical understanding (Figure 1).

**TABLE 1:** Comparison of IRP monitoring and IFA monitoring in infection prevention and control during URS.

	IRP monitoring	IFA monitoring
Pathophysiological Role	Primary physical driving force for IFA	Material vehicle for bacterial/endotoxin entry into bloodstream

Monitoring Metric	Instantaneous hydrostatic pressure (mmHg or cmH_2_O)	Cumulative volume of fluid absorbed (mL)

Pathophysiological Meaning	Reflects instantaneous risk of intrarenal backflow	Reflects cumulative pathogenic load absorbed by the patient; theoretically directly correlates with total bacteria/endotoxins entering the circulation

Key Monitoring Technologies	• UAS with pressure channel: preserves lumen, measures at UPJ	• End‐tidal ethanol monitoring: sensitive, noninvasive, requires special breathing circuit
	• Integrated sensor ureteroscope: high accuracy, high cost	• ESMS: real‐time, noninvasive, also monitors blood loss
	• Pressure wire/ureteral catheter: good accuracy, occupies working channel	• Volumetric/gravimetric balance: simple, error‐prone, clinically limited
		• Isotope/chemical tracers: accurate, complex, mainly research use

Primary Clinical Value	Active Prevention: Provides real‐time feedback for immediate intervention (e.g., adjusting irrigation, activating suction) to mitigate the driving force before significant fluid backflow occurs.	Sentinel Warning: Monitors cumulative absorption to assess realized risk; may signals need for salvage or intensified therapy before overt infection manifests.

Main Limitations	• Cannot account for contribution of operative duration to cumulative risk	• Inherent lag; significant absorption indicates pathogenic substances have already entered the circulation
	• Cannot detect changes in tissue permeability due to mucosal injury	• Outcome monitoring; cannot interrupt the absorption process itself
	• Single‐point measurement may miss localized high pressure in a calyx	

Current Level of Clinical Evidence/Applicability	Moderate: Multiple URS studies with intelligent pressure control systems demonstrating reduced postoperative fever/SIRS; IRP thresholds (≤ 30–40 mmHg) supported by clinical and animal data. Commercially available technologies.	Limited: Mechanistic plausibility supported by animal models and related procedures (mini‐PCNL, TURP); lacks URS‐specific validated thresholds and prospective intervention trials. Mainly a research tool at present.

Guidance for Clinical Decisions	Maintaining IRP below the backflow threshold is a fundamental preventive measure, especially in high‐risk patients (e.g., preoperative infection).	IFA quantification aids comprehensive infection risk assessment, guiding escalation of antimicrobial therapy or enhanced postoperative monitoring when indicated.

*Note:* IRP, intrarenal pressure; URS, ureteroscopy; UPJ, ureteropelvic junction.

Abbreviations: ESMS, endoscopic surgical monitoring system; IFA, irrigation fluid absorption.

### 5.1. The Actionable Value of IRP Monitoring

IRP monitoring’s core value is immediacy and actionability. As the driving force variable (*P*_renal_(*t*)), IRP changes precede actual absorption. Real‐time feedback allows immediate surgeon intervention—reducing irrigation pump pressure, repositioning the scope, opening drainage or activating negative pressure suction—when pressure approaches or exceeds the 30 mmHg backflow threshold. Intelligent pressure control devices can automatically maintain IRP within a preset safe range [10–12]. This closed‐loop feedback mitigates the driving force at its source.

However, sole IRP monitoring cannot account for cumulative operative time (*T*) or increased tissue permeability (*K*) from laser or instrument trauma. In complex cases, even with IRP below the safe limit, prolonged surgery and mucosal disruption may allow continuous low‐pressure leakage of contaminated fluid. Most current methods provide single‐point readings, potentially missing higher compartmentalized calyceal pressures and creating a false‐negative sense of security. The 30 mmHg safe threshold is a statistical average; for an individual with thin parenchyma or severe infection, the venous backflow threshold may be significantly lower. Actively maintaining low IRP via monitoring is a necessary but not sufficient condition for infection prevention.

### 5.2. The Infection Risk Warning Value of IFA Monitoring

IFA monitoring (e.g., ethanol breath test or ESMS) provides cumulative data integrated over procedure time. Theoretically, for infected stones, IFA volume highly correlates with the quantity of absorbed bacteria and endotoxins, reflecting true pathogenic load. IFA is the final product integrating pressure, time, effective absorption area and tissue injury. All risk factors converge into the IFA value. Accordingly, IFA may be a better indicator of cumulative pathogen entry and a potentially stronger predictor of SIRS and urosepsis risk; however, this has not been confirmed by prospective interventional studies. If real‐time IFA values become available, an elevated IFA might prompt specific intraoperative interventions, such as reducing irrigation pressure, increasing suction, shortening or staging the procedure and intensifying postoperative monitoring. In high‐risk patients, a predefined IFA value could hypothetically guide the escalation of antibiotic therapy or early goal‐directed fluid resuscitation, but these strategies remain purely theoretical and require validation. This warning potential could guide clinicians to upgrade antibiotic regimens, extend monitoring or implement aggressive fluid resuscitation before full‐blown infection manifests, but appropriate thresholds for triggering such actions are currently undefined.

## 6. Limitations of the Current Evidence

This review has several limitations that should be acknowledged. First, as a narrative review, the literature selection was not systematic and is subject to potential bias. Second, much of the mechanistic evidence linking IRP and IFA to infection derives from animal models (e.g., porcine URS) or related procedures such as mini‐PCNL; direct prospective evidence in human URS cohorts remains sparse. Third, while the proposed absorption kinetics model is mechanistically sound, it has not been validated in a clinical URS setting. Fourth, and most importantly, there are currently no clinically validated IFA thresholds that predict SIRS or urosepsis after URS. The potential value of IFA‐guided management, such as antibiotic escalation or early postoperative intervention, remains a hypothesis to be tested in future prospective studies. Fifth, although IFA monitoring may provide a more comprehensive assessment of absorbed pathogenic load, it does not directly prevent absorption; it primarily serves as a warning tool without interrupting the absorption process itself. Therefore, the conclusions regarding IFA’s predictive superiority over IRP should be interpreted cautiously and are primarily hypothesis‐generating at this stage.

## 7. Conclusion and Future Perspectives

IRP and IFA constitute the core dimensions of safety monitoring during URS. IRP, as the fluid dynamic driving force, enables real‐time pressure feedback for active prevention, preemptively mitigating backflow at its source. IFA, as the cumulative outcome of pressure, time and tissue permeability, may provide a more objective assessment of pathogenic load and holds potential for improved predictive capabilities for postoperative infection risk, though this remains to be proven. Intelligent pressure‐controlled irrigation systems may become standard, typically monitoring IRP via pressure‐sensing UASs or sensor‐integrated URSs with automated negative pressure suction. IFA monitoring may offer significant predictive value but requires specialized equipment and awaits clinical validation of actionable thresholds. The ideal future platform would integrate high‐precision IRP monitoring, real‐time IFA monitoring (once technically mature and clinically validated), an intelligent irrigation pump and automated negative pressure suction, with the aim of automatically balancing surgical efficacy and patient safety based on integrated data. Prospective studies are needed to define IFA thresholds for infection prediction and to determine whether IFA‐guided interventions can measurably reduce postoperative infectious morbidity.

## Funding

This study was supported by the National Natural Science Foundation of China (No. 82360156), the Cuiying Scientific and Technological Innovation Program of The Second Hospital and Clinical Medical School, and Lanzhou University (No. CY2025‐LC‐A01).

## Conflicts of Interest

The authors declare no conflicts of interest.

## Data Availability

Data sharing is not applicable to this article as no datasets were generated or analysed during the current study.
